# New Nanohybrid Based on Hydrolyzed Polyacrylamide and Silica Nanoparticles: Morphological, Structural and Thermal Properties

**DOI:** 10.3390/polym12051152

**Published:** 2020-05-18

**Authors:** María C. Ruiz-Cañas, Henderson I. Quintero, Laura M. Corredor, Eduardo Manrique, Arnold R. Romero Bohórquez

**Affiliations:** 1 Grupo de Investigación en Química Estructural, Parque Tecnológico Guatiguará, Universidad Industrial de Santander, A.A. 678, Piedecuesta 681011, Colombia; 2 Instituto Colombiano del Petróleo, ECOPETROL S.A., Piedecuesta 681011, Colombia; henderson.quintero@ecopetrol.com.co (H.I.Q.); laura.corredor@ecopetrol.com.co (L.M.C.); eduardo.manrique@ecopetrol.com.co (E.M.)

**Keywords:** nanomaterials, nanohybrids, synthetic polymers, nanoparticles, characterization of polymers

## Abstract

In this study, a set of advanced characterization techniques were used to evaluate the morphological, structural, and thermal properties of a novel molecular hybrid based on silica nanoparticles/hydrolyzed polyacrylamide (CSNH-PC1), which was efficiently obtained using a two-step synthetic pathway. The morphology of the nanohybrid CSNH-PC1 was determined using scanning electron microscopy (SEM), dynamic light scattering (DLS), and nanotracking analysis (NTA) techniques. The presence of C, N, O, and Si atoms in the nanohybrid structure was verified using electron dispersive scanning (EDS). Moreover, the corresponding structural analysis was complemented using powder X-ray diffraction (XRD) and attenuated total reflection-Fourier transform infrared spectroscopy (ATR-FT-IR). The covalent bond between APTES-functionalized SiO_2_ nanoparticles (nSiO_2_-APTES), and the hydrolyzed polyacrylamide (HPAM) chain (MW ≈ 20.10^6^ Da) was confirmed with high-resolution X-ray spectroscopy (XPS). Finally, the thermal properties of the nanohybrid were evaluated by using thermogravimetric analysis (TGA) and differential scanning calorimetry (DSC). The results showed that the CSNH-PC1 has a spherical morphology, with sizes between 420–480 nm and higher thermal resistance compared to HPAM polymers without modification, with a glass transition temperature of 360 °C. The integration of these advanced characterization techniques implemented here shows promising results for the study and evaluation of new nanomaterials with multiple applications.

## 1. Introduction

Nanotechnology refers to the manipulation of matter at the nanoscale (1–100 nm) at the atomic, molecular, and supramolecular levels [1]. Materials at the nanoscale have shown significantly improved chemical, physical, and biological properties relative to the same materials at a larger scale [2]. Nowadays, nanotechnology has an enormous impact in different fields and industries. Some of them are food and agricultural [3,4], oil and gas [2,5,6,7,8,9], paint and coating [10,11], construction [12], medicine [13], cosmetics [14], and wastewater treatment [15]. Nanotechnology in the upstream oil and gas industry has been used to improve the performance of fracturing fluids, improve cement properties, reduce the loss of drilling fluids and enhance wellbore stability, prevent scale deposition, and improve oil recovery. Boul & Ajayan (2020) [16] and Alsaba et al. (2020) [17] present a detailed description of these applications. In the downstream process, the applications include the use of nano-catalysts for refining and petrochemical processes and nano-membranes for enhanced gas separation [6].

**Figure 1:**
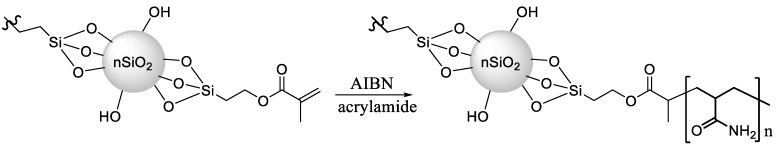
Synthesis of polyacrylamide grafted silica nanoparticle (PAAGS).

Recently, nanotechnology has shown new opportunities in the development of advanced organic-inorganic nanocomposites. Polymer nanocomposites consist of polymer or copolymer matrices containing nanofillers or nanostructured particles [18]. In this sense, nanohybrids are compounds or materials in which two or more molecular scaffolds are covalent bonding or fused, and at least one of the motifs that make it up have nanosize dimensions. Before preparing nanocomposites or nanohybrids, the filler surface (often hydrophilic) must be functionalized to avoid the aggregation of nanoparticles (NP) in the polymer matrix. Physical interaction and chemical reaction are the methods employed to functionalize the filler surface. In the physical interaction, the filler is modified with a surfactant or a polymer through hydrogen bonding, electrostatic, and Van der Waals forces [19]. In the chemical method, the filler is modified with coupling agents or by grafting polymer onto the filler surface (Figure 1) [20]. The polymer grafting can be performed using two techniques, namely “grafting to” and “grafting from.” In the “grafting to” method, a preformed polymer is attached to the filler surface. The “grafting from” is achieved by the in-situ polymerization of monomers on the filler surface [21]. Nanotubes, layered silicates, metal oxides, nanoparticles, and semiconductors have been used as fillers [19,22,23]. However, the silica particles have received more attention due to their high surface area, ordered structure, relatively low cost, and ease of surface modification [24,25].

Many experimental studies have shown that the performance of composite material could be significantly improved when combined or copolymerized with functional monomers containing nano-SiO_2_. Composite materials containing nano-SiO_2_, such as polyethylene terephthalate [26], styrene-butadiene rubber [27], polyaniline [28], polyimide [29], and nylon 6 [30], have been used to improve the properties of the polymer matrices. On the other hand, the nano-SiO_2_ functional monomer (NSFM) has been introduced into AM (acrylamide)/AA(acrylic acid) copolymers, aiming to obtain a higher tolerance to salinity, temperature and shear resistance [25,31,32,33]. The improved properties of these nanocomposites are attributed to the effects of physical adsorption, hydrogen bonding, Si–O and C–Si bonding [34,35,36]. The latter is explained because with this technique, denser polymers with a lower hydrodynamic radius and similar molecular weight can be manufactured.

Several techniques have been used to characterize the properties of nanomaterials, such as shape, size, degree of aggregation, structure, surface charge, and chemistry [37]. The limitations and strengths of each characterization technique complicate the choice of the most suitable method. Consequently, a combination of different techniques is often required to characterize the nanomaterials accurately. In this study, a comprehensive characterization approach was implemented to evaluate the morphological, structural, and thermal properties of a nano-silica/hydrolyzed polyacrylamide hybrid. To the best of our knowledge, there are no literature investigations that report a nanohybrid with the same properties as those described in this work. The results showed that this approach is a promising alternative to assess the properties of nanohybrids for different applications.

## 2. Materials and Methods

### 2.1. Materials

The SiO_2_ NPs were prepared by the Stöber method using tetraethoxysilane (TEOS, 98%, Sigma-Aldrich, St. Louis, MO, USA), ethanol (EtOH, 96%, Merck Millipore, Burlington, MA, USA) and ammonium hydroxide (28–30 wt% solutions of NH_3_ in water, Merck Millipore, USA). The chemicals used to modify the SiO_2_ NPs were 3-aminopropyltriethoxysilane (APTES, 99%, Sigma-Aldrich, USA) and hydrolyzed polyacrylamide (HPAM) with a 25%–35% hydrolysis degree and a molecular weight of 20 × 10^6^ Dalton.

### 2.2. Nanohybrid Synthesis

The SiO_2_ NPs synthesis was performed based on the classic method described by Stöber and Fink [2,38]. First, 1 mL (4.48 mmol) of tetraethyl orthosilicate (TEOS) was added to an ammonium hydroxide solution in ethanol (1:5 ratio). The dispersion obtained was stirred for three hours at 90 °C, and the corresponding NPs were recovered by centrifugation and dried in a conventional oven for 24 h at 90 °C. The previously obtained SiO_2_ NPs were efficiently modified using APTES following the procedure for amino-functionalization proposed by Chen et al. (2009) [39]. Once the nSiO_2_-APTES was purified and characterized, 2 g of this material was dispersed with vigorous stirring in a THF/water solution. The pH of the solution was adjusted to 5–6 using a sulfuric acid solution (0.1 M). Then, the HPAM powder was slowly added to the mixture. When the reaction was over, the corresponding nanohybrid CSNH-PC1 (Core-Shell Nanohybrid Hydrolyzed Polyacrylamide - Nanosilica) was recovered by centrifugation and washed three times with 20 mL of 2-propanol and dried at 60 °C in a conventional oven for 24 h.

### 2.3. Characterization

The size and morphology of the nanohybrids were characterized through field emission gun scanning electron microscopy (FEG-SEM) (QUANTA FEG 650 model, Thermo Fisher Scientific, Waltham, MA, USA) at a high vacuum and an accelerating voltage of 25 kV. The images of the nanohybrid morphology were obtained from the Everhart–Thornley (ETD) and backscattered electron detectors. The samples were placed on metal stubs with carbon adhesive tape. The samples were coated with gold with Quorum 150 ES coating equipment (Quorum Technologies Ltd., Lewes, UK) due to their low conductivity. An energy-dispersive spectroscope (EDS) (EDAX Apolo X, Ametek, INC., Berwyn, PA, USA) with a resolution of 126.1 eV (in. Mn Kα) and an accelerating voltage of 25 kV was used to identify the elemental composition of the nanohybrids.

Dynamic light scattering (DLS) tests were carried out with a Zetasizer Nano ZS90 equipped with a 633 nm He-Ne laser (Malvern Instruments, Herrenberg, Germany) at an angle of 90°. The particle size was evaluated by dispersing the sample in deionized (DI) water at 100 ppm for nSiO_2_-APTES and 1000 ppm for CSNH-PC1. The samples were analyzed in a glass cell with a path length of 10 mm. The measurements were made in a 4.65 mm position from the cuvette wall with an automatic attenuator and at a controlled temperature of 30 °C with an equilibrium time of 180 s. Fifteen runs of 10 s were performed with three repetitions for each sample. The uncertainty in the DLS results is 3.2 nm of the reported value.

The hydrodynamic radius of CSNH-PC1 and the nSiO_2_-APTES was determined by using the nanoparticle tracking analysis (NTA) technique, which was performed with a Nanosight NS300 (Malvern Panalytical, Amesbury, UK) equipped with a sCMOS camera and a 532 nm green laser. The samples were prepared in DI water at a concentration of 100 and 500 ppm. All measurements were performed at 25 °C. The samples were analyzed for 60 s with manual shutter and gain settings in standard mode with five analyses and 25 frames per second (FPS). This technique allows for the real-time visualization of the Brownian movement of the particles in the dispersion medium. The uncertainty in NTA results ranged from approximately 9.3% to 10% of the reported value.

The X-ray diffraction (XRD) pattern, used for the structural analysis, was recorded with a Bruker D-8 A25 DaVinci X-ray diffractometer (D8 Advance, Bruker, Billerica, MA, USA) with CuKα radiation and a LynxEye detector at 40 kV voltage, 40 mA current, 0.6 mm divergence slit, and 2.5° primary and secondary Soller slits. The scan was performed in the range of 2° to 70° with step-by-step scanning over 2θ angles. The NPs were placed on polymethyl methacrylate (PMMA) sample holders with Si center using the zero-background technique. In contrast, the polymer and the nanohybrid were mounted using the frontal filling technique.

The structural characterization of all samples was performed by infrared spectroscopy (ATR-FTIR) using a Bruker Tensor 27 FTIR spectrometer (Alpha, Bruker, USA). The FTIR spectra were collected from 4000–600 cm^−1^ using a total attenuated reflectance (ATR) platinum cell.

Surface chemical binding energies of the nanohybrid and the SiO_2_ NPs were determined using X-ray photoelectron spectroscopy (XPS, SPECS, Berlin, Germany). The XPS experiments were recorded using the XPS/ISS/UPS surface characterization platform. The platform was equipped with a PHOIBOS 150 2DDLD energy analyzer. The pressure in the chamber was approximately 1 × 10^−9^ Pa. An X-ray source of monochromatized AlKα (FOCUS 500) operated at 100 W was used in the measurements. The passing energy of the hemispheric analyzer was set at 100 eV for the broad spectra and 30 eV for the high-resolution spectra. The surface charge compensation was controlled using a flood gun (FG 15/40-PS FG 500), which was operated at 58 μA–2.5 eV. During the final measurement, the C1s region was re-recorded to verify the evolution of the surface charge of the samples. The results were analyzed with the CasaXPS program (Casa Software Ltd., Teignmouth, Devon, England) using the SPECS Prodigy-ACenteno library provided with the response sensitivity factor (RSF). The spectra binding energy scale was correlated, taking as reference the C1s peak at 284.8 eV.

The nanohybrid and the HPAM thermal properties were analyzed by thermogravimetry using a TA2050 TGA analyzer (TA Instruments, INC., New Castle, DE, USA). For the measurements, a mass of 5 mg of sample was heated from 25 to 800 °C at a nitrogen flow of 25 mL/min and a heating rate of 10 °C/min. Thermogravimetric analysis (TGA) allows for the determination of the organic matter content and the degree of functionalization in the nanohybrid. The thermal stability of the new nanomaterial was evaluated based on the ASTM E2550-17 standard (ASTM, 2007). The glass transition temperature of the nanohybrid was determined by using a TAQ25 differential scanning calorimetry (DSC) Analyzer (TA Instruments, INC., USA). For the measurements, a mass of 8 mg was heated from −10 °C to 500 °C at a heating rate of 3 °C/min in a nitrogen atmosphere (at a flow rate of 25 mL/min).

## 3. Results and Discussion

### 3.1. SEM Results

**Figure 2:**
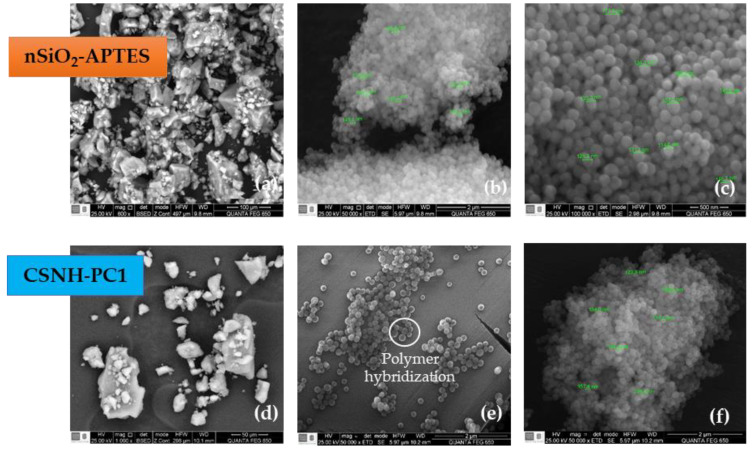
SEM micrographs of (**a**–**c**) nSiO_2_-APTES at 600×, 50,000× and 100,000×, respectively, and (**d**–**f**) CSNH-PC1 at 1000×, 50,000× and 50,000× (2).

SEM images of the CSNH-PC1 and nSiO_2_-APTES are presented in Figure 2. It is observed that the nSiO_2_–APTES have a near-spherical morphology and size of 141 nm (Figure 2a–c). On the other hand, the SEM images for the nanohybrid (Figure 2d–f) clearly show a well-formed structure; on its surface, the nanoparticles are attached to the polymer at specific sites, corresponding to the hybridization area of the polymer.

**Figure 3:**
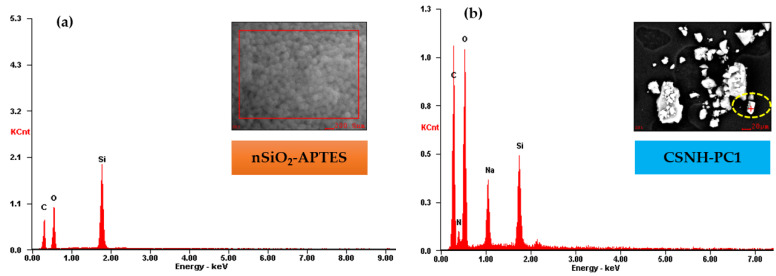
EDS results of (**a**) nSiO_2_-APTES and (**b**) CSNH-PC1.

The EDS results of nSiO_2_-APTES and the nanohybrid are shown in Figure 3. The presence of Si, O, and Na (from sodium acrylate) on the CSNH-PC1 surface confirms the hybridization of the HPAM and the nSiO_2_-APTES.

Table 1 shows that after the chemical attachment of the polymer onto the NPs surface, the Si content in the surface content element of CSNH-PC1 decreased from 22.20 wt% to 5.41 wt% compared to the nSiO_2_-APTES. On the contrary, the surface element content of C, O, and especially N increased in the nanohybrid (CSNH-PC1), clearly suggesting the chemical bonding of the HPAM onto the silica NPs.

### 3.2. DLS Results

**Figure 4:**
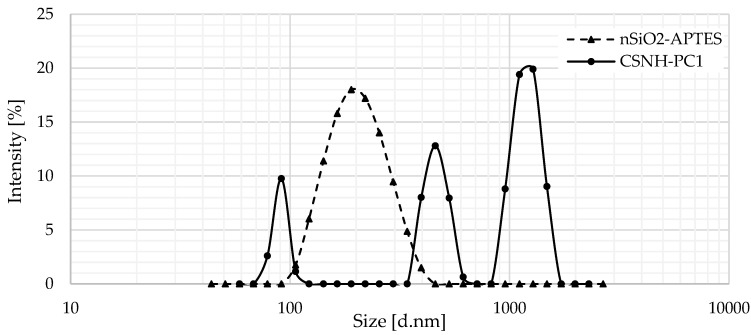
Size distribution by the intensity of nSiO_2_-APTES and CSNH-PC1.

Figure 4 presents the DLS results of nSiO_2_-APTES dispersed in distilled water (DI). It is observed that the NP size distribution is larger than the obtained with the SEM (Figure 2), which was attributed to the interaction of the NPs with the dispersed media (DI) [40]. The size distribution is monomodal, with an average size of 200 nm.

The DLS results for the nanohybrid show a tri-modal size distribution (Figure 4). The peak around 100 nm (nanometric scale) is assigned to the unmodified NPs present in the sample. The peaks between 420 and 480 nm and 1200 to 1400 nm correspond to the CSNH-PC1 particles and particle aggregates formed from intermolecular associations of the HPAM chains by hydrogen bonding, respectively.

### 3.3. Hydrodynamic Radius by NTA

**Figure 5:**
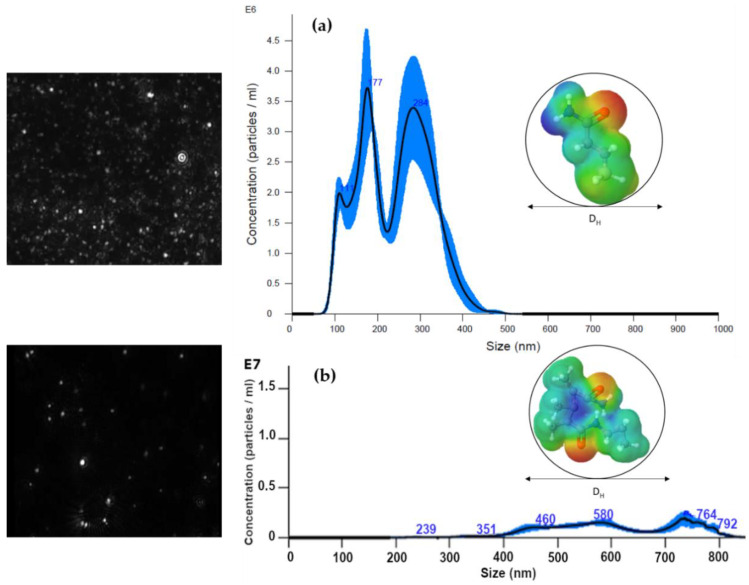
Size distribution by nanoparticle tracking analysis (NTA) of (**a**) nSiO_2_-APTES and (**b**) CSHN-PC1. Colors represent atoms: Grey = carbon; Blue = Nitrogen; White = Hydrogen; Yellow = Sulfur; and Red = Oxygen.

The results of the NTA analysis of the nSiO_2_-APTES are shown in Figure 5a. It is observed that the size distribution is bimodal, which was attributed to the presence of unmodified and modified SiO_2_ NPs in the aqueous solution. The average size of nSiO_2_-APTES is 240 nm, which is consistent with the DLS results.

Comparing the nSiO_2_-APTES (Figure 5a) with the nanohybrid (Figure 5b), the latter shows an increase in polydispersity. This can be explained due to the anionic nature of the HPAM chains. Moreover, the particle size of the nanohybrid is bigger than that of the SiO_2_-APTES. For this reason, the size distribution is shifted to the right, and the nanohybrid particles exhibit less Brownian motion. Additionally, the video frames of Figure 5b show two size range structures at 460 nm and 764 nm. NTA is a powerful characterization technique that complements DLS, and it is particularly valuable for analyzing polydisperse nanosized particles and nanohybrid aggregates [41].

### 3.4. X-Ray Diffraction (XRD) Results

**Figure 6:**
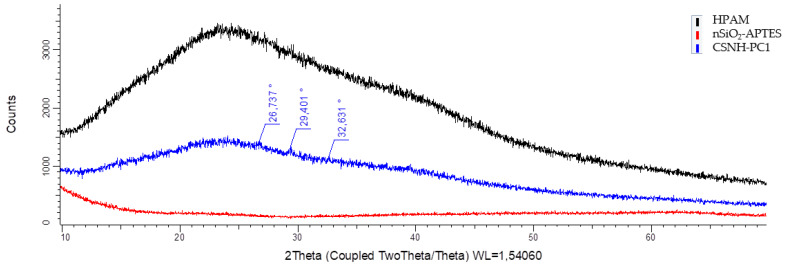
X-ray diffraction patterns of the hydrolyzed polyacrylamide (HPAM), nSiO_2_-APTES, and CSNH-PC1.

The successful formation of organic-inorganic hybrid materials is demonstrated by comparing the XRD patterns of precursors and nanohybrids [42]. Figure 6 shows the diffractogram of nSiO_2_-APTES, CSNH-PC1, and HPAM. The XRD patterns of the nSiO_2_-APTES show the presence of an amorphous halo around 23°, which is assigned to the amorphous SiO_2_ NPs [43,44]. These results suggest that the NPs did not change their amorphous arrangement as a result of the functionalization process [45].

Besides, the peaks at 26.7°, 29.4°, and 32.6° in 2*θ*; in the nanohybrid curve show a partial ordering due to the attachment of the nSiO_2_-APTES with the polymer chains. As depicted in the diffractogram, the nanohybrid shows an amorphous structure. This partial rearrangement tends to the formation of crystals in the structure, which gives greater strength to the bonds. Finally, the formation of the nanohybrid is evidenced by a considerable structural change. The partial arrangement confirms the attachment between the NPs and the HPAM chains.

### 3.5. ATR-FTIR Results

**Figure 7:**
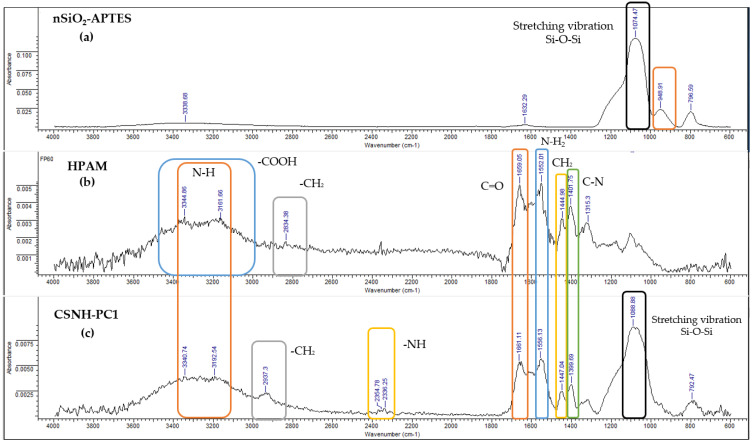
Infrared (IR) spectra of (**a**) nSiO_2_-APTES, (**b**) HPAM and (**c**) CSNH-PC1.

The FTIR spectra of nSiO_2_-APTES, HPAM, and CSNH-PC1 are compared in Figure 7 to verify the attachment of the polymer to the nSiO_2_-APTES surface. The characteristic peaks that confirm the nanohybrid formation are [31,33]:

- 3340 cm^−1^ (–NH stretching vibration and –OH stretching vibration) [46];
- 2937 cm^−1^ (–CH_2_ stretching vibration);
- 1661 cm^−1^ (C=O stretching vibration);
- 1399 cm^−1^ (C–N stretching vibration);
- 1088 cm^−1^ (Si–O–Si asymmetric stretching vibration) and;
- 792 cm^−1^ (Si–O–Si symmetric stretching vibration).

Also, the peak at 2354 cm^−1^ represents the presence of secondary amide in the nanohybrid structure, confirming the covalent bonding between the nSiO_2_-APTES and the HPAM.

### 3.6. X-Ray Spectroscopy (XPS) Analysis

**Figure 8:**
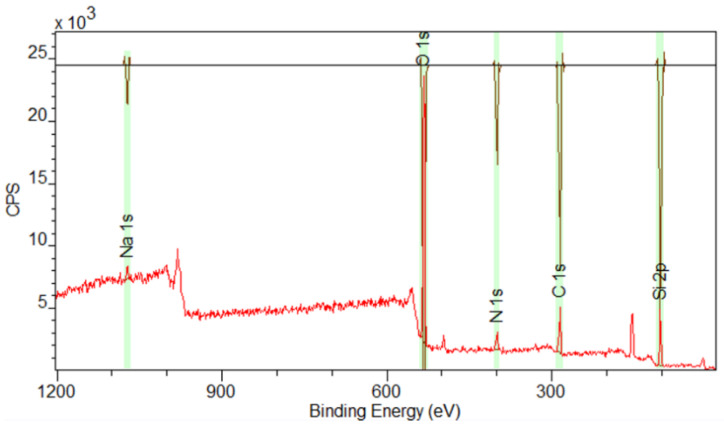
X-ray photoelectron spectroscopy (XPS) spectra of CSNH-PC1.

The surface compositional data of the CSNH-PC1 determined by XPS is present in Figure 8. The adjustment was made concerning carbon pollution in C1s at 284.8 eV. The XPS results of the CSNH-PC1 surface confirm the hybridization of the HPAM and the nSiO_2_-APTES with the presence of C1s, N1s, C1s, Na1s, and Si2p in the regions according to the library and Relative Sensitivity Factors (R.S.F) [47].

The deconvolution of the main spectra in the regions in high-resolution, and the central level spectra of C1s, O1s, N1s, and Si2p for nSiO_2_-APTES and CSNH-PC1 are presented in Table 2. It was performed using the Lorentzian adjustment method, which uses the binding energy, asymmetry of the principal peak, and intensity relationship between the main peaks as adjustment parameters. This is consistent with the NIST (National Institute of Standards and Technology, Gaithersburg, MD, USA) database [47,48] and the studies published by Zienkiewicz-Strzałka et al. (2018) [49] and Burg, et al. (2002) [50].

**Figure 9:**
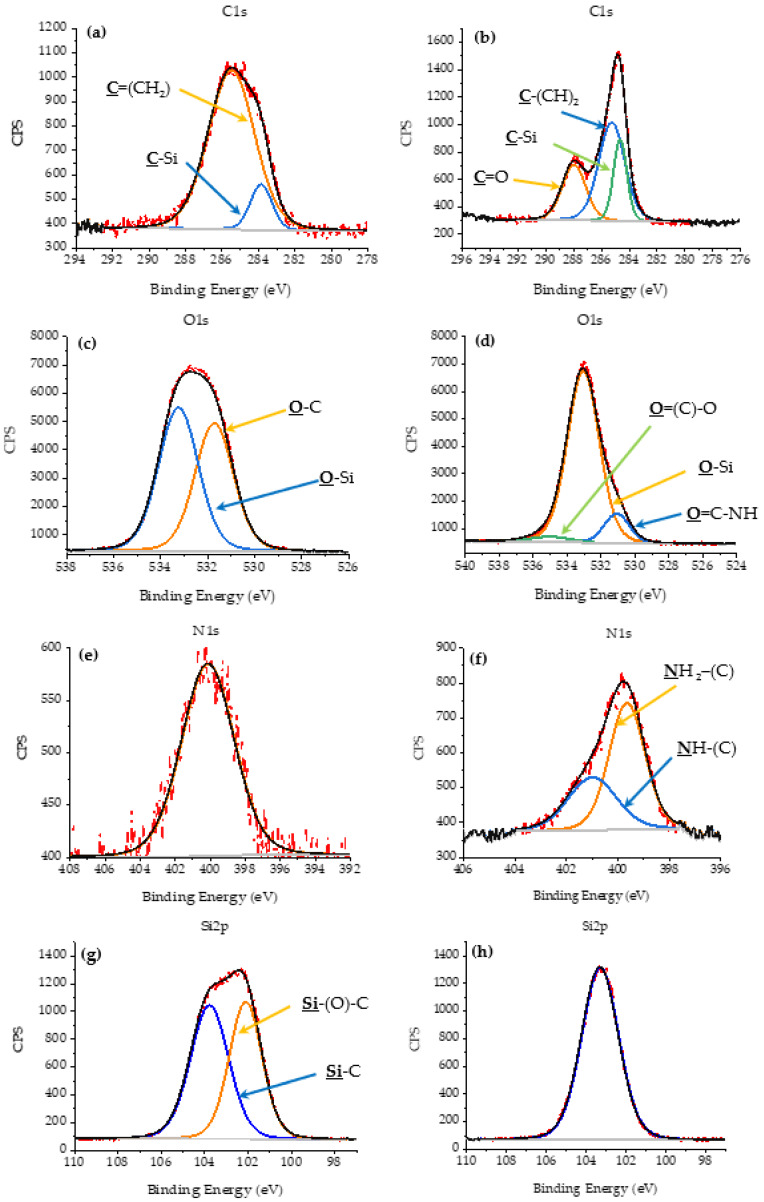
High-resolution X-ray photoelectron spectroscopy (XPS) spectra of (**a**) C1s for nSiO_2_-APTES, (**b**) C1s for CSNH-PC1, (**c**) O1s for nSiO_2_-APTES, (**d**) O1s for CSNH-PC1, (**e**) N1s for nSiO_2_-APTES, (**f**) N1s for CSNH-PC1, (**g**) Si2p for nSiO_2_-APTES and (**h**) Si2p for CSNH-PC1.

In the case of C1s spectra (Figure 9a,b), the carbon contamination was attributed to signals collected from the adhesive carbon tape used as a substrate in the XPS analysis. In these spectra, different signals attributed to the functional groups belonging to the functionalized NPs and nanohybrid (CSNH-PC1) are identified. The carbon-containing species identified were C–C at 285.39 eV, C–Si at 284.38 eV, and C=O around 287.6 eV [49].

The peaks of the O1 spectrum (Figure 9d) that correspond to the O=C–O, O=C–NH, and O–Si species (associated with the carboxylic acids, amides, and the covalent bond with silica in the nanohybrid) were observed at 534.42, 531.14, and 533.02 eV, respectively.

The peak binding energy, in the high-resolution spectrum of N1s, is observed at 399.65 and 400.11 eV for the CSNH-PC1 and the nSiO_2_-APTES, respectively (Figure 9e,f). It can be assigned to the contribution of a primary amide bond. The appearance of a new region at 400.99 eV corresponds to the secondary amide bond generated between the NPs and the polymer, according to the NIST database [48]. Besides, the peak enlargement indicates the change between the number of chemical bonds that contribute to the shape.

In the high-resolution spectrum of Si2p for the nSiO_2_-APTES (Figure 9g), two main peaks appear at 103.78 and 102.12 eV, which are associated with Si–O–C [46] and Si–C species. These peaks are not observed in the nanohybrid (Figure 9h). The peak for Si–O–C was identified at 1003.8 eV because it is the bond that prevails in depth throughout the system. The difference in energy between the peaks is more than 1 eV.

Accordingly, high-resolution spectra obtained by the XPS technique shows the functional groups corresponding to the covalent bond between the polymer and the NP, emphasizing the formation of a secondary amide. To assess the thermal behavior of the hybrid CSNH-PC1, TGA was performed, as will be described in the following section.

### 3.7. Thermal Properties—TGA Results

**Figure 10:**
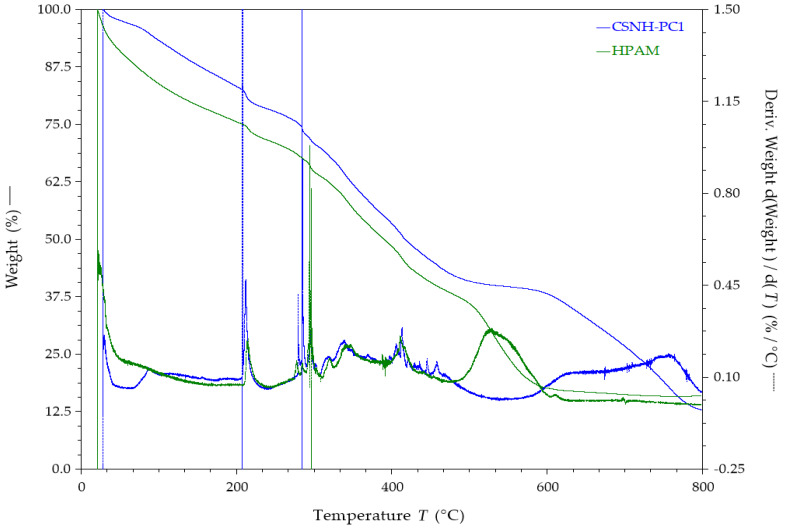
Thermograms of nSiO_2_-APTES and CSNH-PC1 (heating rate of 10 °C/min in a nitrogen atmosphere).

Figure 10 shows the TGA profile of the CSNH-PC1 and HPAM. Three stages of weight loss were observed in the thermogram of both samples. The first stage occurred below 200 °C, which was attributed to the remaining adsorbed water or volatile solvents present in each sample. The weight loss in this zone was 24.3% for HPAM and 16.5% for CSNH-PC1. The second stage was observed from 200 °C to 400 °C, which was assigned to the thermal decomposition of the amide and carboxylate groups of the polymer. The weight loss in this step was 30.43% for HPAM and 29.76% for CSNH-PC1. The final stage (>400 °C) was attributed to the decomposition of the C–C bonds from the HPAM backbone [51].

### 3.8. Differential Scanning Calorimetry (DSC) Characterization Results

**Figure 11:**
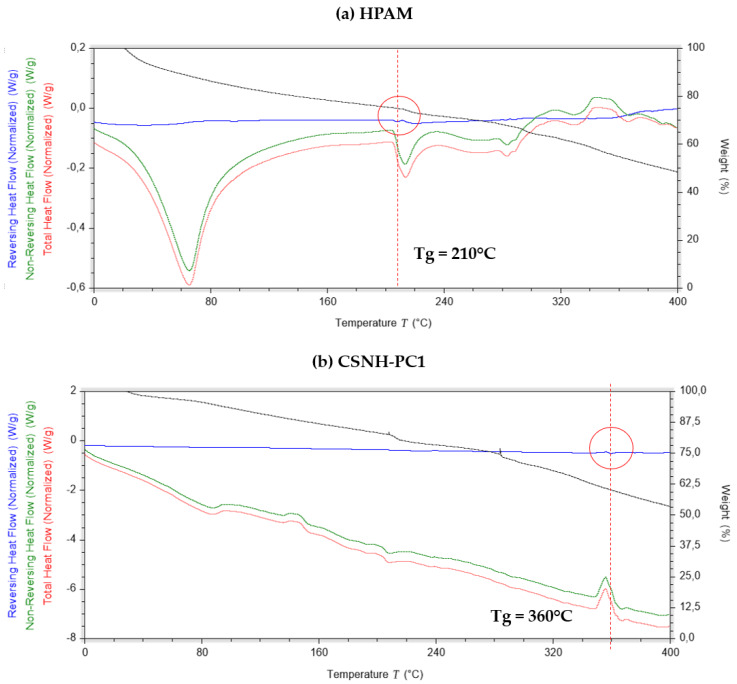
Differential scanning calorimetry (DSC) curves of HPAM (**a**) and CSNH-PC1 (**b**) (heating rate of 3 °C/min in a nitrogen atmosphere).

It is known from the literature that the glass transition temperature (Tg) of a polymer strongly depends on the mobility of its chains, and it can be changed by the addition of a solid filler [52,53,54,55,56]. If there are strong attractive forces between filler and polymer, the Tg increases because the adsorption of polymer chains on the filler surface decreases its mobility. Figure 11 shows that the Tg of the CSNH-PC1 (360 °C) is higher than that of the HPAM (210 °C). This effect is attributed to the reduction of the mobility of the HPAM on the nSiO_2_-APTES surface by three dominant mechanisms (i) the chemical covalent bonding of the polymer chains to the NPs surface, (ii) the HPAM chains’ confinement onto the NPs surface [56], and (iii) the increment of the degree of crosslinking of the polymer chains [57,58]. Consequently, the inclusion of the nanoparticles in the polymer matrix improved the thermal stability of the HPAM.

## 4. Conclusions

A novel nanosilica/polyacrylamide hybrid was successfully prepared using a two-step synthetic pathway. XPS and ATR-FTIR confirmed the covalent bond between the nSiO_2_-APTES and the HPAM chains. The presence of C, N, O, and Si atoms in the nanohybrid structure was verified with EDS analysis. The SEM, DLS, and NTA techniques revealed that the particle size of the nSiO_2_-APTES was around 240 nm. For the nanohybrid, two size range structures were observed (around 460 nm and 1000 nm). Finally, the DSC measurements indicated that the incorporation of nanosilica into the polymer matrix positively impacts the thermal properties of the HPAM by increasing its glass transition temperature from 210 °C to 360 °C.

The present study demonstrates that the use and integration of advanced characterization techniques, such as those proposed in this study, represents an important contribution to research and development in the area of new materials for multiple applications.

## Figures and Tables

**Table 1:** Weight and atomic percentage for nSiO_2_-APTES and CSNH-PC1 according to EDS analysis.

Element	nSiO_2_-APTES		CSNH-PC1	
	Wt%	At%	Wt%	At%
C	36.29	47.16	37.79	45.92
N	0.50	0.50	8.95	9.32
O	41.51	40.50	41.60	37.95
Si	22.20	12.34	5.14	2.67
Na			6.52	4.14

**Table 2:** Binding energy values of the main functional groups.

Energy Level	Functional Groups	CSNH-PC1	nSiO_2_-APTES	NIST Database	Zienkiewicz et al.	Burg et al.
C1s	C–(CH_2_)	285.3	285.5	285.4		285.0
	C–(Si)	284.7	283.9	284.4		
	C=(O)	288.0		287.6		287.9
O1s	O=(C)–O	534.4		535.1		532.0
	O=(C)–NH	531.1	531.7	530.7		531.2
	O–(Si)	533.0	533.2	532.3	532.7	530.3
N1s	NH_2_–(C)	399.7	400.1	399.2		399.9
	NH–(C)–O	401.0		400.0		
Si2p	Si–(O)–C	103.3	103.8	102.9	103.5	
	Si–(C)		102.1	101.7	101.2	
